# Determinants of Disease Penetrance in *PRPF31*-Associated Retinopathy

**DOI:** 10.3390/genes12101542

**Published:** 2021-09-28

**Authors:** Samuel McLenachan, Dan Zhang, Janya Grainok, Xiao Zhang, Zhiqin Huang, Shang-Chih Chen, Khine Zaw, Alanis Lima, Luke Jennings, Danial Roshandel, Sang Yoon Moon, Rachael C. Heath Jeffery, Mary S. Attia, Jennifer A. Thompson, Tina M. Lamey, Terri L. McLaren, John De Roach, Sue Fletcher, Fred K. Chen

**Affiliations:** 1 Centre for Ophthalmology and Visual Science, The University of Western Australia, Nedlands, WA 6009, Australia; smclenachan@lei.org.au (S.M.); xiaozhang2626@gmail.com (X.Z.); ZhiqinHuang@lei.org.au (Z.H.); DanialRoshandel@lei.org.au (D.R.); rachaelheathjeffery@lei.org.au (R.C.H.J.); MaryAttia@lei.org.au (M.S.A.); tina.lamey@health.wa.gov.au (T.M.L.); Terri.McLaren@health.wa.gov.au (T.L.M.); John.DeRoach@health.wa.gov.au (J.D.R.); 2 Ocular Tissue Engineering Laboratory, Lions Eye Institute, Nedlands, WA 6009, Australia; dana@lei.org.au (D.Z.); Shang-ChihChen@lei.org.au (S.-C.C.); KhineZaw@lei.org.au (K.Z.); luke.jennings3490@hotmail.com (L.J.); SangYoonMoon@lei.org.au (S.Y.M.); 3 Centre for Molecular Medicine and Innovative Therapeutics, Murdoch University, Murdoch, WA 6150, Australia; janya.grainok@pyctx.com (J.G.); Alanis.Lima@murdoch.edu.au (A.L.); 4 Australian Inherited Retinal Disease Registry and DNA Bank, Department of Medical Technology and Physics, Sir Charles Gairdner Hospital, Nedlands, WA 6009, Australia; Jennifer.Thompson3@health.wa.gov.au; 5 Centre for Neuromuscular and Neurological Disorders, The University of Western Australia, Nedlands, WA 6009, Australia; 6 Department of Ophthalmology, Royal Perth Hospital, Perth, WA 6000, Australia; 7 Department of Ophthalmology, Perth Children’s Hospital, Nedlands, WA 6009, Australia

**Keywords:** retinitis pigmentosa, RP11, *PRPF31*, *CNOT3*, *MSR1*, non-penetrance, rod-cone dystrophy, induced pluripotent stem cells, retinal pigment epithelium, retinal organoid

## Abstract

Retinitis pigmentosa 11 (RP11) is caused by dominant mutations in *PRPF31*, however a significant proportion of mutation carriers do not develop retinopathy. Here, we investigated the relationship between *CNOT3* polymorphism, *MSR1* repeat copy number and disease penetrance in RP11 patients and non-penetrant carriers (NPCs). We further characterized *PRPF31* and *CNOT3* expression in fibroblasts from eight RP11 patients and one NPC from a family carrying the c.1205C>T variant. Retinal organoids (ROs) and retinal pigment epithelium (RPE) were differentiated from induced pluripotent stem cells derived from RP11 patients, an NPC and a control subject. All RP11 patients were homozygous for the 3-copy *MSR1* repeat in the *PRPF31* promoter, while 3/5 NPCs carried a 4-copy *MSR1* repeat. The *CNOT3* rs4806718 genotype did not correlate with disease penetrance. *PRFP31* expression declined with age in adult cadaveric retina. *PRPF31* and *CNOT3* expression was reduced in RP11 fibroblasts, RO and RPE compared with controls. Both RP11 and NPC RPE displayed shortened primary cilia compared with controls, however a subpopulation of cells with normal cilia lengths was present in NPC RPE monolayers. Our results indicate that RP11 non-penetrance is associated with the inheritance of a 4-copy *MSR1* repeat, but not with *CNOT3* polymorphisms.

## 1. Introduction

Inherited retinal diseases (IRDs) are the most common cause of blindness in the working-age population [1,2]. Retinitis pigmentosa (RP) is the most common form of IRDs accounting for almost half of the Australian national IRD registry [3]. Characterised by initial night vision impairment followed by progressive symmetrical constriction of the visual field, RP contributes to 64% of IRD-related blindness certification at a mean age of 48 years [1]. The Online Mendelian Inheritance in Man (OMIM) has listed RP genotype sequentially from RP1 to RP91. RP11, caused by mutations in the *pre-mRNA processing factor 31* (*PRPF31*) gene (OMIM #600138) accounts for 5–10% of all the autosomal dominant forms of RP worldwide [4,5,6]. RP11 typically presents as a progressive rod-cone dystrophy, with a highly variable age of onset within and between affected families. There are two unique features of *PRPF31*-related disease. First, the carriers of pathogenic *PRPF31* mutations can exhibit non-penetrance despite the manifestation of a severe RP phenotype in other family members carrying the same mutation [7,8]. Second, mutations in this gene lead to non-syndromic RP despite the ubiquitous expression of *PRPF31* and its important role in splicing in all cell types. A deeper understanding of the molecular mechanisms underlying non-penetrance and non-syndromic manifestations of RP11 are essential for the development of safe and effective therapy for RP11.

The *PRPF31* gene encodes the pre-mRNA processing factor-31 protein, which forms part of the U4/U6/U5 small nuclear ribonucleoprotein (snRNP) subunits of the spliceosome, a large RNP complex central to the process of pre-mRNA splicing [9,10,11,12,13]. Pre–mRNA splicing is an essential step in gene expression whereby non–coding intronic sequences are removed from the transcribed pre–mRNA molecule to produce a mature mRNA transcript carrying the protein coding sequence. Several explanations have been put forward for the non–penetrance of *PRPF31* mutations in asymptomatic carriers. Studies in immortalized lymphoblastoid cell lines (LCLs) harboring *PRPF31* mutations demonstrated a link between *PRPF31* expression and disease penetrance, with increased expression in non-penetrant carriers (NPCs) compared with affected RP11 patients [14,15,16]. Furthermore, genetic linkage studies showed disease non-penetrance was associated with inheritance of modifier sequences located within the 19q13.4 interval that contains the *PRPF31* gene [14,17]. These observations led to the theory that disease non-penetrance was influenced by a penetrance-modifying sequence within 19q13.4 located in a gene proximal to *PRPF31*. In 2012, Venturini et al. proposed that this modifier gene was *CNOT3* [18], which encodes a subunit of the Carbon Catabolite Repression-Negative On TATA-less (CCR4-NOT) transcriptional complex, a master regulator of global gene expression [19]. These authors identified a single nucleotide polymorphism (rs4806718, T/T), located within intron 17 of *CNOT3* that was significantly associated with disease non-penetrance in two RP11 pedigrees [18]. Following on from their previous work, Rose et al. later published strong evidence indicating that a significant proportion of NPCs in RP11 families may also be attributed to the inheritance of a highly expressive, unaffected *PRPF31* allele [20]. In that study, they identified the minisatellite repeat element 1 (*MSR1*) within the *PRPF31* promoter, and further showed that a 4-copy repeat element was associated with higher expression of the *PRPF31* allele in LCLs, while inheritance of a 3-copy repeat led to lower expression. In patients with *PRPF31* mutations, haploinsufficiency of PRPF31 protein is thought to lead to a global increase in non-productive splicing, affecting the expression of a large number of retinal genes [21,22]. These global effects on splicing cause defects in cilia length in photoreceptor progenitor cells (PPCs) and retinal pigment epithelium (RPE) produced from induced pluripotent stem cells (iPSC) derived from patients with RP11 [21]. These cellular phenotypes may serve as a suitable disease model for studying the non-penetrant and non-syndromic features of RP11.

We recently described the clinical features of RP11 from eight unrelated families [23,24]. Here, we investigated the role of *CNOT3* and the 4-copy *MSR1* repeat in RP11 non-penetrance in a cohort of 29 individuals carrying eight different pathogenic *PRPF31* mutations from eight unrelated families. We examined the relative expression of *PRPF31* and *CNOT3* in dermal fibroblasts as well as induced pluripotent stem cell (iPSC)-derived retinal organoids (RO) and retinal pigment epithelium (RPE) and characterized the primary cilia in iPSC-derived RPE.

## 2. Materials and Methods

### 2.1. Human Tissues

The collection and use of human tissues in this study was approved by the University of Western Australia (RA/4/1/7916, RA/4/20/5717) and the Sir Charles Gairdner Hospital Human Research Ethics Committees (2001–053, 2012–090). Written consent was obtained from the patients and all procedures were carried out in accordance with the National Health and Medical Research Council of Australia National Statement on Ethical Conduct in Human Research (2007–Updated 2018) and the Declaration of Helsinki. Retinal tissues from deceased adult human donors were obtained from the Lions Eye Bank (Lions Eye Institute, Nedlands, Western Australia).

### 2.2. Clinical Assessment

Patients with genetically confirmed *PRPF31* mutation underwent comprehensive routine ophthalmic assessment including detailed medical and ocular history, best-corrected distance visual acuity (BCVA), complete slit lamp eye examination, functional assessment and structural multimodal retinal imaging (see below). A clinical diagnosis of RP was made based on history of impaired night vision, funduscopic findings of attenuated retinal vessels, bone spicules and retinal atrophy, and the observation of a profound reduction in dark-adapted full-field ERG response, where available. The NPCs were determined based on lack of symptoms of nyctalopia or constricted visual field, absence of typical fundus features on retinal examination or multimodal imaging and a normal visual field if available, despite the presence of a known disease associated *PRPF31* variant. Detailed clinical descriptions and pedigrees for the eight families in our studies have previously been published [20,21].

### 2.3. Genetic Diagnosis and Pathogenicity Assessment 

DNA samples were sourced, processed and stored as detailed previously [25]. Genetic screening of RP11 patients was previously reported [23,24]. Variants are described in accordance with Human Genome Variation Society recommendations [26]. Nucleotide one corresponds to the A of the start codon, ATG. The OMIM accession number and gene reference sequence utilised are #600138 and NM_015629.3, respectively. Pathogenicity was assessed as previously reported [27] and interpreted in accordance with current guidelines proposed by the American College of Medical Genetics and Genomics and the Association for Molecular Pathology [28], and Jarvik and Browning [29].

### 2.4. CNOT3 Polymorphism and Copy Number of MSR1

For genotyping the *CNOT3* rs4806718 single nucleotide polymorphism, DNA was extracted from blood samples or cultured fibroblasts using the FlexiGene DNA kit (Qiagen, Hilden, Germany). PCR amplification was followed by Sanger sequencing. Primers used in this study are listed in Supplementary Table S1. The number of copies of the *MSR1* repeat element adjacent to the *PRPF31* promoter was assessed using PCR analysis. Primers were designed to amplify gDNA across the reference *MSR1* region (hg 19 coordinates chr19:54617996–54619393), as previously reported by Rose et al. [20].

### 2.5. Dermal Fibroblast Culture

Human dermal fibroblasts were obtained from the Western Australian Retinal Disease Study Biobank and cultured in DMEM medium supplemented with 10% fetal calf serum and antibiotic-antimycotic (ThermoFisher, Waltham, MA, USA). Media was changed twice per week. Fibroblasts were harvested for RNA analysis across three separate passages for cells from each patient. For reprogramming and gene expression measurements, early passage fibroblasts were used (<p8). 

### 2.6. Cadaveric Adult Human Retina

Adult human eyes were obtained from 7 male and 4 female donors aged 22–64 years from the Lions Eye Bank, Western Australia. The mean time (±SD) from death to retinal tissue harvesting was 18 (±5) h. The retina was dissected from the posterior eye cup and stored at −80 °C in RNAlater. Total RNA was isolated from whole retinae using TRIzol Reagent (ThermoFisher, Waltham, MA, USA).

### 2.7. Induced Pluripotent Stem Cells

Fibroblasts from a 28 year old male RP11 patient and a 54yo female NPC from the same RP11 family (c.1205C>A variant) were previously reprogrammed into iPSC [30]. For this study, one additional iPSC line from this RP11 patient and NPC were utilized. Additional iPSC lines were generated from two unrelated RP11 patients, one carrying a deletion of exons 2–8 and the other carrying the c.267del variant in *PRPF31*. For controls, we utilized iPSC lines previously generated from a 24 year old healthy control subject. Human iPSC were cultured in feeder-free conditions, on geltrex (ThermoFisher, Waltham, MA, USA) coated culture plates in mTeSR1 medium (StemCell Technologies, Vancouver, BC, Canada), as previously described [30]. 

### 2.8. Retinal Organoid Differentiation

Human iPSC colonies were dissociated into small pieces by mechanical dissociation after 2–4 min of incubation with EDTA buffer (0.5 mM EDTA and 30 mM NaCl in DPBS without Ca^2+^ or −Mg^2+^). Cell clusters were seeded into suspension culture plates and cultured in DMEM/F-12 medium (ThermoFisher, Waltham, MA, USA) containing MEM Non-Essential Amino Acids Solution (NEAA, ThermoFisher, Waltham, MA, USA), B27 (ThermoFisher, Waltham, MA, USA), human recombinant IGF-1 (StemCell) and KnockOut Serum Replacement (KOSR, ThermoFisher, Waltham, MA, USA). The KOSR concentrations were gradually decreased from 20% for the first 5 days, to 15% until day 12 and to 10% until day 35). From day 35, KOSR was removed from the media. Half media changes were performed every day, with full media changes every second–third day, up to 200 days of culture.

### 2.9. RPE Differentiation

In addition to RO, the abovementioned protocol for RO differentiation resulted in the production of highly pigmented ‘RPE organoids’ that are largely comprised of RPE cells. These RPE organoids were removed from RO cultures after 6–8 weeks of differentiation and transferred into geltrex-coated 24-well plates with RPE media containing DMEM/F-12 medium with 1x NEAA, 1x B27, human recombinant 10 ng/mL IGF-1 (Stem Cell Technologies) and 4% KOSR (ThermoFisher, Waltham, MA, USA). Media was changed every third day. RPE organoids formed pigmented cell clusters within 4–6 weeks (RPEp0). RPE clusters were dissociated by incubation with TrypLE Express Enzyme for 20 min at 37 °C, and passaged into new geltrex coated wells using a split ratio of 1:5. Pure RPE monolayers were usually established after two passages. RPE monolayers were used for experiments at passage 3–4, 4 weeks after plating.

### 2.10. Quantitative PCR

Total mRNA was isolated using TRIzol Reagent (ThermoFisher, Waltham, MA, USA) and cDNA was synthesized using the RT^2^ First Strand Kit (Qiagen, Hilden, Germany). qPCR reactions were prepared using RT^2^ SYBR Green qPCR Mastermixes (Qiagen, Hilden, Germany) and performed using the CFX Connect Real-Time System (BioRad Laboratories, Hercules, CA, USA). Data was analyzed using the ΔΔCT method. Gene expression values were normalized against *GAPDH* expression. For fibroblast analysis, gene expression values were calculated as fold-change compared with expression level in fibroblasts derived from a 24-year old male control subject. For retinal organoid analysis, gene expression values were calculated as fold-change compared with the mean expression value derived from three cadaveric human retinal samples from working aged adults (28–60 years old). For RPE analysis gene expression values were calculated as fold-change compared with control iPSC-derived RPE. Primers used in this study are listed in Supplementary Table S1.

### 2.11. Immunostaining

Cells were fixed with 4% paraformaldehyde for 15 min at 37 °C, washed, then permeabilized using 0.3% Triton X-100 in phosphate buffered saline (PBS) for 15 min. The cells were then incubated in blocking buffer (5% normal goat serum and 0.3% Triton X-100 (Sigma-Aldrich, St. Louis, MO, USA) in PBS) for 1 h at room temperature. Primary antibodies were diluted in blocking buffer and applied at 4 °C, overnight. Secondary antibodies were diluted in blocking buffer and applied for 2 h at room temperature. Nuclei were stained with DAPI (1 μg/mL). Antibodies and dilution factors used in this study are listed in Supplementary Table S2. Cells were examined using the Olympus BX60 fluorescence microscope and images were captured using Olympus DP-Controller 3.1.1.267 acquisition software (Olympus Corporation, Tokyo, Japan).

### 2.12. Scanning Electron Microscopy

For scanning electron microscopy (SEM), RPE cells were passaged onto Geltrex coated Millicell hanging cell culture inserts with pore size of 0.4 µm (Millipore Sigma, Burlington, MA, USA). After 4 weeks of culture, inserts were removed following Lynn’s method and fixed in 2.5% glutaraldehyde in 0.1 M phosphate buffer (pH 7.4) for 20 min at room temperature. Critical dehydration and platinum coating were performed according to a previously published SEM sample preparation protocol [31]. Finally, SEM samples were visualized using a field emission scanning electron microscope (Carl Zeiss Supra 55 FESEM, Zeiss, Oberkochen, Germany).

### 2.13. Cilia Length Analysis

Primary cilia in RPE monolayers were labeled by immunostaining for ARL13B followed by counterstaining with DAPI. Cells were imaged using the Nikon Instruments A1 Confocal Laser Microscope. For primary cilium length measurements, maximum projection intensity images were generated from confocal stacks using NIS Elements Viewer (Version 4.11.0, Laboratory Imaging) and cilia lengths measured using Image J64 software (National Institute of Health, Bethesda, MD, USA). Mean cilia lengths were compared using the student *t*-test, with *p* < 0.05 considered significant. Comparison of DAPI stained nuclei counts and cilia counts in each field indicated >98% of cells displayed a primary cilium. Unciliated cells were not included in our analysis.

## 3. Results

### 3.1. Demographics of RP11 Patients

A total of 29 individuals from eight unrelated RP11 families contributed DNA for *CNOT3* and *MSR1* genotyping. Five individuals from four families had no symptoms or signs of RP. Affected individuals had a mean ± SD age of 14 ± 11 years at the onset of symptoms. Clinical phenotypes have been previously published [23,24].

### 3.2. The CNOT3 rs4806718 Polymorphism Does Not Correlate with RP11 Disease Penetrance

Amongst the five NPCs, three (60%) carried the C/T genotype whilst two (40%) carried the T/T genotype at the rs4806718 locus. Amongst the 24 affected RP11 patients, 16 (66%) carried the C/T genotype, four (17%) carried the T/T genotype and four (17%) carried the C/C genotype at the rs4806718 SNP (Table 1). The higher prevalence of the T/T rs4806718 genotypes in NPCs compared to affected RP11 patients (40% vs. 17%) was not statistically significant (χ^2^ = 1.37, *p =* 0.24) suggesting this locus does not strongly influence disease penetrance in our RP11 cohort. 

### 3.3. The 4-Copy MSR1 Repeat Is Associated with RP11 Disease Penetrance

Genotyping of the *MSR1* repeat element demonstrated the inheritance of a homozygous 3-copy *MSR1* repeat element (3/3 genotype) in all affected patients, while three of the five NPCs carried a 4-copy repeat element (3/4 genotype) (Table 1). Together, these results demonstrate disease non–penetrance is associated with the inheritance of *PRPF31* alleles containing 4-copy *MSR1* repeats in their promoter region.

### 3.4. PRPF31 and CNOT3 Expression Is Reduced in RP11 Patient Fibroblasts

**Figure 1:**
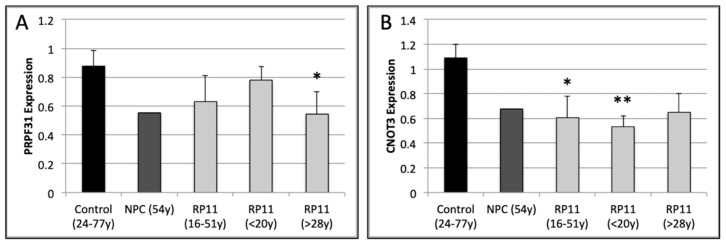
PRPF31 and CNOT3 expression was measured in fibroblasts derived from eight affected RP11 patients (RP, light grey bars) and a non-penetrant carrier (NPC, dark grey bars) carrying the c.1205C>A variant in PRPF31 as well as three unrelated control subjects (Con, black bars), by qPCR. Mean PRPF31 and CNOT3 expression values were calculated for each fibroblast line from RNA samples obtained from 2–6 different passages. Mean expression values for PRPF31 (**A**) and CNOT3 (**B**) were calculated for the healthy control subject group (24–77 years old, *n =* 3) and the affected RP11 patient group (16–51 years old, *n =* 8). The RP11 group was further subdivided into teenage (<20 years old, *n =* 3) and adult (>28 years old, *n =* 5) patients. Statistical significance was calculated using a two-tailed *t*-test (* *p* < 0.05, ** *p* < 0.01, compared with control group).

Dermal fibroblast cultures were derived from eight patients with RP11 and one asymptomatic non-penetrant carrier (NPC) from one RP11 family carrying the c.1205C>A variant [23], as well as three unrelated control subjects, aged 24, 46 and 77 years old (yo). *PRPF31* and *CNOT3* mRNA expression was measured in fibroblast cDNA samples by quantitative PCR (qPCR) analysis. The mean level of *PRPF31* expression across the RP11 patient group (*n =* 8) was not significantly different from the control group (*n* = 3, *p =* 0.055). Interestingly, mean *PRPF31* expression levels in fibroblasts from RP11 patients in their teens (<20yo, *n =* 3) were similar to controls, while expression in fibroblasts derived from adult patients (>28yo) was significantly reduced compared with control fibroblasts (*n =* 5, *p* = 0.0173, Figure 1A). 

*CNOT3* expression was significantly reduced in the RP11 patient group (*p* = 0.0170) compared with the control group (Figure 1B). Within the RP11 patient group, teenaged patients showed significantly lower levels of *CNOT3* expression than controls (*n =* 3, *p* = 0.0095). Mean *CNOT3* expression levels were similarly reduced in adult RP11 patients, however this reduction was not significant compared with controls (*p* = 0.0717). Together, these results suggest that *PRPF31* and *CNOT3* expression is reduced in fibroblasts carrying the c.1205C>A variant. 

### 3.5. PRPF31 mRNA Expression Is Reduced with Age in Cadaveric Adult Retina

**Figure 2:**
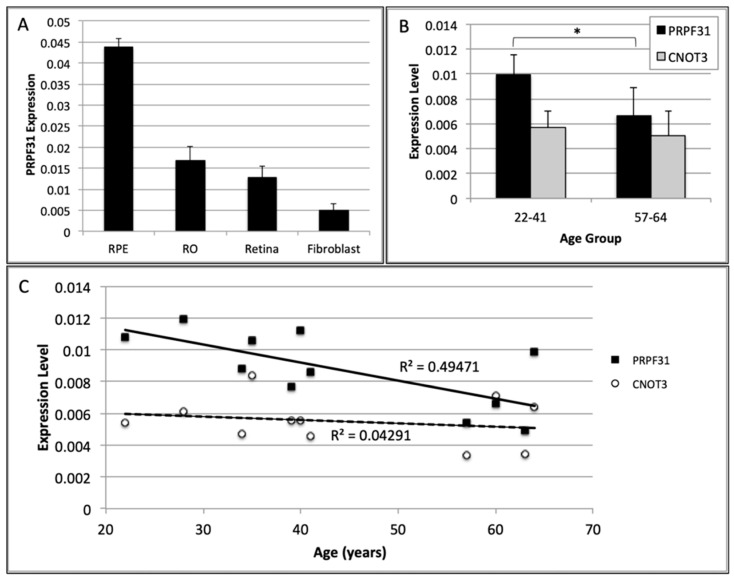
(**A**) *PRPF31* expression was measured in control iPSC-derived RPE cells (RPE) and retinal organoids (RO), adult human retinal tissues (3 donors, aged 22–64) and control fibroblasts (3 donors aged 24–77) by qPCR. Gene expression values were expressed as fold-change compared with *GAPDH*. Error bars indicate standard deviation. (**B**) *PRPF31* and *CNOT3* expression was measured in adult human retinal samples by qPCR. Older donors (aged 57–64, *n =* 4) showed significantly reduced *PRPF31* expression compared with younger donors (aged 22–41, *n =* 7, * *p* < 0.05). (**C**) Linear regression analysis of *PRPF31* and *CNOT3* expression in adult human retinal tissues from eleven donors showed a decline in *PRPF31* expression with increasing age, while *CNOT3* expression showed no correlation with age.

*PRPF31* mRNA levels were higher in adult human retina, iPSC-derived retinal organoids (ROs) and RPE cells compared with fibroblasts, with the highest levels detected in RPE monolayers (Figure 2A). In retinal tissues obtained from donated human eyes, *PRPF31* expression was found to decrease with advancing donor age (Figure 2B), with older donors (57–64 years old) showing significantly reduced *PRPF31* expression compared with younger donors (22–41 years old, *p =* 0.0189). Linear regression analysis demonstrated a moderate negative correlation between *PRPF31* expression and age (R^2^ = 0.495, Figure 2C). In contrast, *CNOT3* expression was similar in retinal tissues from old and young subjects and did not correlate with age (R^2^ = 0.043, Figure 2B–C).

### 3.6. PRPF31 mRNA Expression Is Reduced in RP11 Retinal Organoids

**Figure 3:**
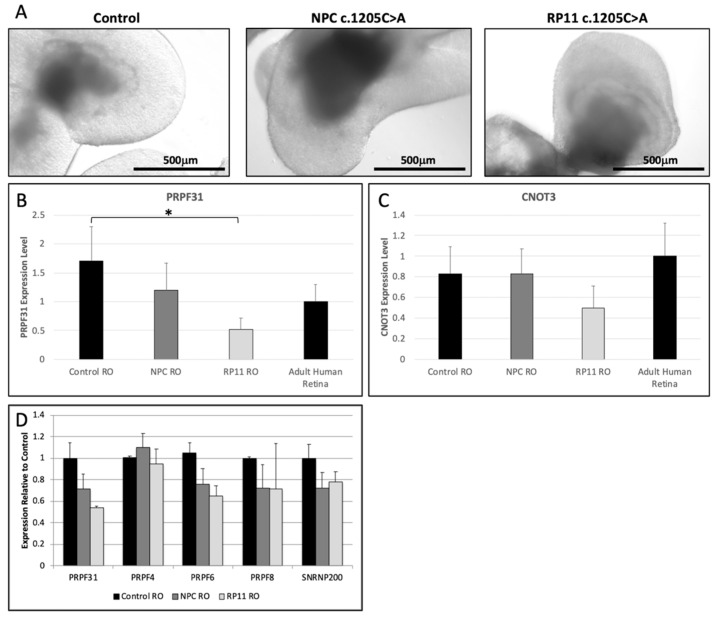
Retinal organoids (RO) were differentiated from iPSC derived from an RP11 patient and a non–penetrant carrier (NPC) carrying the c.1205C>A variant in *PRPF31*, and a healthy control subject (**A**). *PRPF31* (**B**) and *CNOT3* (**C**) expression was normalized to *GAPDH* and expressed as fold-change compared with values obtained from adult human retinal tissues (3 donors, aged 22–64). Each bar represents the mean from 3–4 retinal organoids from each subject. Error bars indicate standard deviation. Statistical significance was calculated using a two-tailed *t*-test (* *p* < 0.05). Expression of the spliceosome genes *PRPF31*, *PRPF4*, *PRPF6*, *PRPF8* and *SNRNP200* was measured in day 35 retinal organoid cultures (**D**) derived from control subjects (black bars), the NPC (dark grey bars) and the RP11 patient (light grey bars) by qPCR. Error bars indicate standard deviation.

To examine *PRPF31* expression in retinal cells, we differentiated iPSC derived from the RP11 patient and NPC carrying the c.1205C>A mutation or a control subject into RO (Figure 3A). After six months of differentiation, expression of the retinal markers *PAX6*, *OTX2*, *CRX* and *RCVRN* was upregulated in RO compared with undifferentiated iPSC (Supplementary Figure S1A). Additionally, RT-PCR analysis demonstrated the retinal specific transcript variant of *BBS8* was expressed in RO cultures (Supplementary Figure S1B). 

*PRPF31* expression was significantly reduced in RP11 patient RO (*p =* 0.0231) compared with control RO. In contrast, NPC RO showed an intermediate *PRPF31* expression level that was not significantly different from control RO (*p =* 0.2821) or RP11 patient RO (*p =* 0.0875) (Figure 3B). No significant differences in *CNOT3* expression were detected between control, NPC and RP11 patient RO (Figure 3C). 

### 3.7. Expression of Spliceosome Genes Is Reduced in RP11 RetinalOorganoids

In addition to reduced *PRPF31* expression, early ROs (day 35) derived from the RP11 patient and the NPC displayed reduced expression of the spliceosome genes *PRPF6*, *PRPF8* and *SNRNP200* compared with control ROs. In contrast, *PRPF4* showed similar levels of expression in RP11 patient, NPC and control ROs (Figure 3D).

### 3.8. PRPF31 and CNOT3 Expression Is Reduced in RP11 iPSC-Derived RPE

**Figure 4:**
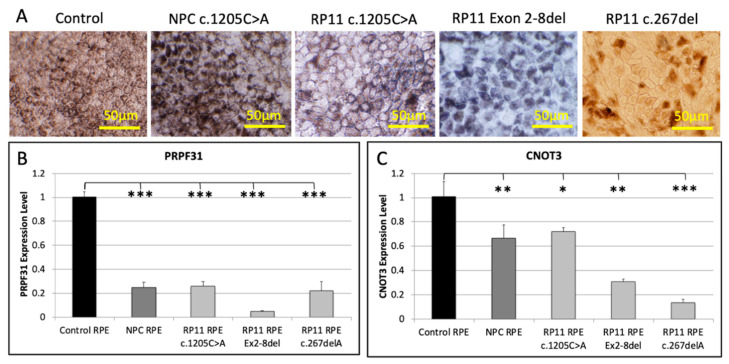
RPE monolayers were differentiated from iPSC derived from a control subject, a non-penetrant carrier (NPC) and RP11 patient carrying the c.1205C>A mutation, an RP11 patient carrying a deletion in exons 2–8 and an RP11 patient carrying the c.267del mutation (**A**). *PRPF31* (**B**) and *CNOT3* (**C**) expression were normalized to *GAPDH* and expressed as fold-change compared with values obtained from control RPE. Each bar represents the mean from 3 independent RPE culture wells. Error bars indicate standard deviation. Statistical significance was calculated using a two-tailed *t*-test (* *p* < 0.05, ** *p* < 0.01, *** *p* < 0.001).

RPE cells were differentiated from iPSC derived from a control subject, an RP11 patient and an NPC carrying the c.1205C>A variant, an RP11 patient carrying a deletion of exons 2–8 and an RP11 patient carrying the c.267del variant (Figure 4A). RPE cells formed pigmented monolayers comprised of RPE65, bestrophin, MITF, CRALBP and ZO1 immunopositive cells (Supplementary Figure S2A–C). Upregulation of the RPE markers *RPE65* and *BEST1* was demonstrated in cultured iPSC-derived RPE by qPCR (Supplementary Figure S2D). 

*PRPF31* expression was significantly reduced (*p* < 0.001) in RPE derived from the three RP11 patients and the NPC, compared with control RPE, with the patient carrying the exon 2–8 deletion showing the lowest levels (Figure 4B). *CNOT3* expression was also significantly reduced in RP11 (*p* < 0.05–0.001) and NPC RPE (*p* < 0.01) compared with control RPE (Figure 4C). These results demonstrate that expression of *PRPF31* was reduced both in RP11 patient and NPC iPSC-derived RPE cells, suggesting the 4-copy *MSR1* repeat associated with disease non-penetrance leads to increased expressivity of the *PRPF31* allele in neural retinal cells, but not in RPE cells.

### 3.9. Microvilli Densities Are Reduced in RP11 iPSC-Derived RPE

**Figure 5:**
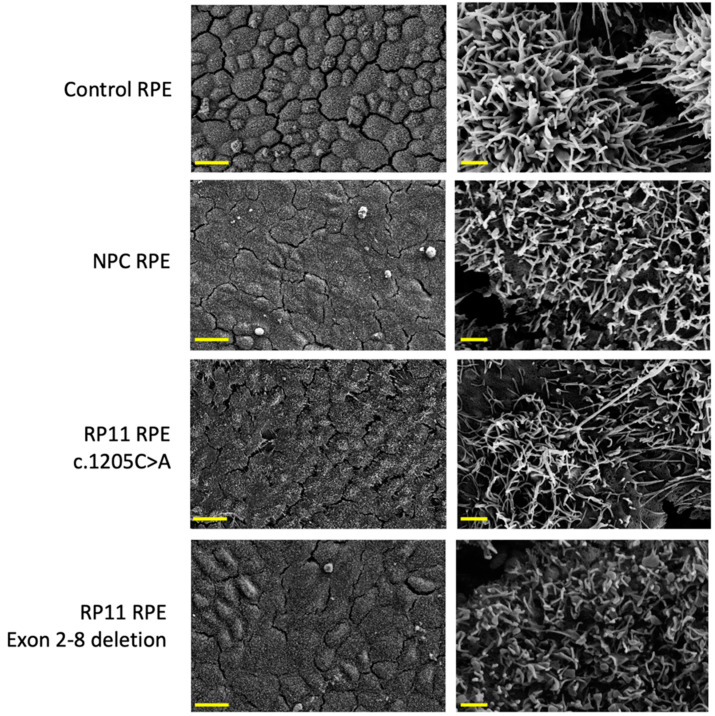
Scanning electron micrographs show RPE monolayers derived from a control subject, a non-penetrant carrier (NPC) and RP11 patient carrying the c.1205C>A mutation and an RP11 patient carrying a deletion in exons 2–8, at 1000× (left panels, scale bar indicates 20 μm) and 30,000× magnification (right panels, scale bar indicates 1 μm).

Ultrastructural analysis of iPSC-derived RPE by scanning electron microscopy revealed differences in cell morphology between controls and RP11 patients. Control RPE demonstrated regularly spaced, polygonal morphology with high surface densities of microvilli. In contrast, RPE derived from NPC and RP11 patient iPSC displayed a flattened morphology, with patches of elongated or hypertrophic cells with reduced microvilli densities on their apical surfaces (Figure 5).

### 3.10. Primary Cilia Length Is Reduced in RP11 iPSC-Derived RPE

**Figure 6:**
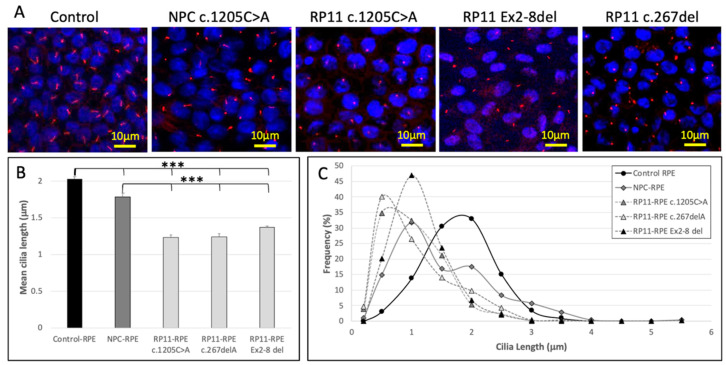
(**A**) Merged fluorescence micrographs show RPE monolayers derived from a control subject, a non-penetrant carrier (NPC) and RP11 patient carrying the c.1205C>A mutation, an RP11 patient carrying the c.267del variant and an RP11 patient carrying a deletion in exons 2–8. RPE was stained with the primary cilia marker, ARL13B (red signal) and the nuclear marker DAPI (blue signal). (**B**) Primary cilia length was significantly shorter in RPE derived from the RP11 patient or the NPC, than in RPE from the unaffected control (*** *p* < 0.001). (**C**) Frequency distributions of primary cilia lengths in RPE derived from control iPSC, the RP11 patients and NPC.

To investigate the effects of reduced *PRPF31* expression on ciliogenesis in iPSC–derived RPE cells, RPE monolayers derived from the healthy control subject, the RP11 patient and NPC carrying the c.1205C>A variant, the RP11 patient carrying the c.267del variant and the RP11 patient carrying the exon 2–8 deletion were labeled with an antibody targeting the primary cilium marker ARL13B. More than 98% of the cells from both control and RP11 iPSC-derived RPE monolayers displayed a primary cilium, however RPE derived from both RP11 patients and the NPC showed increased frequencies of short, ‘stunted’ cilia (Figure 6A). Quantification of cilia length showed significantly shortened cilia in the RP11 patients compared with RPE from the healthy control (*p* < 0.001). The mean length of NPC RPE primary cilia was longer than that measured in RP11 patient RPE (*i* < 0.001), but shorter than primary cilia of healthy control RPE (*p* < 0.001, Figure 6B).

In healthy control RPE primary cilia lengths were normally distributed around a median value of 2 μm. In contrast, the distributions of cilia lengths were skewed towards shorter lengths in RP11 patient RPE, with two patients showing peak frequencies at 0.5 μm and one patient showing peak frequency at 1 μm (Figure 6C). These results indicate the majority of RPE cells derived from RP11 patients displayed stunted cilia. Interestingly, two peaks were observed in the primary cilia length distribution of the NPC RPE, suggesting two populations of cells with median cilia lengths of 1 μm (stunted) and 2 μm (normal) (Figure 6C).

## 4. Discussion

Since the identification of *PRPF31* as the causal gene underlying RP11, multiple hypotheses have been proposed to explain the features associated with this autosomal dominant inherited retinal disease. For example, the retinal specificity of RP11 has been linked to high levels of *PRPF31* expression and increased dependence on alternative splicing in retinal cells [32,33,34]. Another feature of RP11 is the variable penetrance of disease among patients carrying heterozygous *PRPF31* null alleles. Although inheritance of a single *PRPF31* null allele is associated with the development of RP11 in the majority of patients (75–95%, depending on the population studied), most reported RP11 cohorts included individuals who carried the pathogenic mutation but did not develop retinal disease [14,15,17,18,35]. The factors underlying disease non-penetrance in these individuals have been the focus of a number of studies, since their identification could provide novel insights to enhance clinical disease prognostication as well as suggesting new targets for treatment. Given the retinal specificity of *PRPF31* deficiency, it is important that potential mechanisms of non-penetrance uncovered in unaffected cell types are validated in the affected retinal cell types. Currently, only one other study examining the effect of *PRPF31* mutations in iPSC-derived retinal tissues from RP11 patients has been published [21]. Here, we provide the first report characterizing retinal organoids and RPE cells generated from a non-penetrant carrier. In this study, we examined two proposed determinants of non-penetrance, *CNOT3* and *MSR1*, using fibroblasts and iPSC-derived retinal cells from patients with RP11. 

The *CNOT3* gene encodes a subunit of the Ccr4-NOT transcriptional complex, a master regulator of global gene expression [19]. Ccr4-NOT complexes regulate gene expression through modulation of transcription by binding promoter elements, as well as through modulation of RNA stability by binding directly to mRNA [36,37]. *CNOT3* has been proposed to function as a tumor suppressor gene and was found to be mutated at high frequencies in T-cell lymphoblastoid leukemia cells [38], while in colorectal and non-small cell lung cancers, *CNOT3* overexpression was shown to promote proliferation [39,40]. Knockdown of *CNOT3* led to tumor formation in a sensitized *Drosophila melanogaster* model [38] and inflammatory phenotypes in mice [41,42]. In 2012, Venturini et al. demonstrated an inverse relationship between *PRPF31* and *CNOT3* expression in LCLs from six RP11 patients and four NPCs, suggesting the higher *PRPF31* expression observed in NPC LCLs may result from reduced transcriptional repression by CNOT3-containing Ccr-NOT complexes. Supportively, the authors identified a single nucleotide polymorphism (rs4806718), located within intron 17 of *CNOT3*, which was significantly associated with disease non-penetrance in 38 subjects from two RP11 pedigrees. Furthermore, CNOT3 was shown to directly bind to the *PRPF31* promoter, and inhibition of *CNOT3* with siRNA increased *PRPF31* expression in cultured ARPE19 cells [18]. While these results suggested *CNOT3* might regulate *PRPF31* expression in LCLs, the linkage disequilibrium between these two neighboring genes has made it difficult to untangle the effects of the rs4806718 SNP from the possibility that it could be marking a contiguous *PRPF31* allele. Indeed, the authors noted the correlation between inheritance of rs4806718 and disease non-penetrance was ‘not perfect’ and our results in a cohort of 29 subjects from eight families demonstrated the rs4806718 genotypes present in the NPCs were also present in affected RP11 patients, suggesting this SNP does not strongly influence disease penetrance in these families. In this study, we sought to examine whether *CNOT3* expression was correlated with disease penetrance and *PRPF31* expression in an RP11 family by measuring expression levels in fibroblasts as well as iPSC-derived retinal cells. Our results showed both *PRPF31* and *CNOT3* expression were reduced in fibroblasts, RO and RPE derived from affected RP11 patients and an unaffected NPC. In support of our observations, Buskin et al. previously demonstrated mis-splicing of *CNOT3* mRNA in RP11 patient-iPSC derived RPE [21]. Although expression levels were not quantitated, inefficient splicing of *CNOT3* pre-mRNA would reduce levels of mature mRNA due to frameshifting of coding sequences and nonsense mediated decay. Together with the results of Buskin et al. [21], our results suggest *PRPF31* haploinsufficiency leads to mis-splicing of *CNOT3*, resulting in reduced *CNOT3* expression in RP11 patient cells.

In addition to *CNOT3* mis-splicing, Buskin et al. identified genes associated with the spliceosome as one of the most common subsets affected by *PRPF31* haploinsufficiency in RP11 patient derived RO and RPE [21]. Supportively, knockdown of *PRPF31* expression in organotypic adult human retinal cultures was recently shown to cause mis-splicing of transcripts encoding the spliceosome subunits *PRPF3*, *PRPF8*, *PRPF4* and *PRPF19* [22]. Here, we observed decreased expression of *PRPF6*, *PRPF8* and *SNRNP200* in RP11 patient-derived RO compared with RO derived from a healthy control, suggesting splicing defects initially caused by *PRPF31* deficiency may be further amplified through reductions in expression of other components of the splicing machinery. 

Following on from their previous work examining *CNOT3* as a potential modifier of RP11 disease penetrance, Rose et al. published strong evidence indicating that a significant proportion of non-penetrance in RP11 families could be attributed to the inheritance of a highly expressive, unaffected *PRPF31* allele [20]. In that study, the authors identified an *MSR1* repeat element within the *PRPF31* promoter, and further showed that a 4-copy repeat element was associated with higher expression of *PRPF31* in RP11 patient LCLs, while inheritance of a 3-copy repeat led to lower expression. In a cohort of 42 affected RP11 patients and 29 unaffected NPCs, 4-copy repeats were not found in any affected RP11 patients, but were present in almost one third of NPCs. The frequencies of the 4-copy *MSR1* variant of the *PRPF31* allele within different ethnic populations was strongly correlated with the frequencies of non-penetrance measured in RP11 cohorts derived from these populations, suggesting the *MSR1* repeat element is a major determinant of non-penetrance in RP11 [20,43]. In support of these previous observations, we found that a 4-copy *MSR1* repeat element was present in 3/5 unaffected NPCs from different RP11 pedigrees, but was not found in any affected RP11 patients. Consistent with previous observations in LCLs, RO derived from an NPC carrying the 4-copy *MSR1* repeat showed an intermediate level of *PRPF31* expression between RO derived from one of her affected sons and control RO levels. Interestingly, inheritance of the 4-copy *MSR1* repeat element did not correlate with significant differences in *PRPF31* expression in RPE cells derived from the NPC, suggesting the effects of this promoter element could be specific to some cell types, such as neural retinal cells and LCLs. However, further studies with larger numbers of NPC patients will be required to determine the relationship between *MSR1* genotype and expression of the unaffected *PRPF31* allele in NPC retinal cells.

In dermal fibroblasts, reductions in *PRPF31* expression were more pronounced in cells from adult RP11 patients, while cells from teenaged patients had higher levels of *PRPF31* mRNA. Additionally, we demonstrated *PRPF31* expression in the adult human retina significantly decreases with age. Age-related declines in *PRPF31* expression highlight the need for age-matched control subjects in studies performed in patient-derived primary cell cultures. Notably, the NPC studied here was the eldest subject (54yo) in the family and expressed 65% of the *PRPF31* mRNA levels measured in fibroblasts derived from the closest age-matched control (46yo), and almost double the levels of the closest age-matched RP11 patient (51yo) from the same family. These results are consistent with the modest increases in *PRPF31* expression previously reported in NPC LCLs compared with LCLs from affected RP11 patients [18] and suggest that the inheritance of the 4-copy *MSR1* repeat element is associated with increased *PRPF31* expression in NPC fibroblasts, compared with an age-matched RP11 patient carrying homozygous 3-copy repeats. 

Although dermal fibroblasts and LCLs represent accessible cell models for examining the expression of ubiquitously expressed genes such as *PRPF31*, their use in the identification of disease mechanisms is limited by a lack of a retinal cell phenotype. In this study, we confirmed the ciliopathy phenotype previously reported by Buskin et al. in RP11 patient derived RPE cells [21] and further showed the variable penetrance of this phenotype in RPE cells derived from the NPC. Compared with RPE derived from a control subject, RPE cells derived from the unaffected NPC showed similar reductions in *PRPF31* expression levels as RPE cells derived from an affected RP11 patient, however, cilia length distributions in NPC RPE revealed the presence of two populations of RPE cells. Although the majority of cells were affected by a ‘stunted’ cilia phenotype (62% < 1.5 μm), a sizable subpopulation of cells displayed normal length cilia (38% > 1.5 μm). It is possible that these two populations represent RPE cells below and above a critical threshold of *PRPF31* expression. Ultrastructural characterization by SEM supported this conclusion, with patches of flattened, elongated RPE cells interspersed with unaffected cells present in NPC RPE monolayers. It is difficult to predict how defective ciliogenesis in RPE cells in vitro might reflect disease processes in vivo, since RP11 patients are born with functional RPE. It is likely that in the absence of paracrine support from the vasculature, resident immune cells and neural retina, cultured RPE cells are more prone to disturbances in cellular homeostasis than their in vivo counterparts. However, despite these potential limitations, the stunted cilia phenotype in RP11 patient iPSC-derived RPE could provide a useful measure for screening of pharmacological agents with potential for treating RP11 through increasing *PRPF31* expression in RPE cells.

## 5. Conclusions

Together, our results demonstrate reduced *PRPF31* expression in retinal cells derived from RP11 patients was associated with reduced *CNOT3* expression and that *CNOT3* polymorphism did not correlate with non-penetrance. We showed that inheritance of the 4-copy variant of the *MSR1* repeat element was associated with disease non-penetrance. We further demonstrate a ‘stunted’ cilia phenotype in RPE monolayers derived from RP11 patients, with variable penetrance in the NPC. This variable penetrance at the cellular level suggests NPC-derived RPE cells are balanced on the edge of *PRPF31* insufficiency, further suggesting that small increases in *PRPF31* expression may be sufficient to restore splicing defects in haploinsufficient retinal cells.

## Figures and Tables

**Table 1:** RP11 Patient Genotyping.

Patient ID	Group/Age/Sex	*PRPF31* Variant (NM_015629.3)	*PRPF31* *MSR1*	*CNOT3* rs4806718
**Family 1**				
1846	NPC 11 M	c.1205C>A	3/4	T/T
1439	RP11 16 M	c.1205C>A	3/3	T/T
1479	RP11 18 M	c.1205C>A	3/3	C/T
1486	RP11 19 M	c.1205C>A	3/3	C/T
1582	RP11 22 F	c.1205C>A	3/3	C/T
1093	RP11 28 M *	c.1205C>A	3/3	C/T
1341	RP11 31 M	c.1205C>A	3/3	C/T
1576	RP11 35 F	c.1205C>A	3/3	C/C
1299	RP11 35 F	c.1205C>A	3/3	C/T
1485	RP11 38 M	c.1205C>A	3/3	C/T
1063	RP11 51 M	c.1205C>A	3/3	T/T
1374	NPC 54 F *	c.1205C>A	3/4	T/T
1682	RP11 59 M	c.1205C>A	3/3	T/T
**Family 2**				
1332	RP11 29 F	c.267del	3/3	C/C
1506	RP11 34 F	c.267del	3/3	C/T
1651	NPC 40 M	c.267del	3/3	C/T
1150	RP11 51 F *	c.267del	3/3	C/C
1477	RP11 61 M	c.267del	3/3	C/T
1313	RP11 85 F	c.267del	3/3	C/C
**Family 3**				
1681	RP11 16 F	c.772_773delins16 (ins CAACATGCAACATCAT)	3/3	C/T
1757	RP11 18 M	c.772_773delins16 (ins CAACATGCAACATCAT)	3/3	C/T
1816	NPC 56 M	c.772_773delins16 (ins CAACATGCAACATCAT)	3/4	C/T
**Family 4**				
5739	NPC 60 M	Exon 2–3 deletion c.(?_1)_(238+1_239-1)del	3/3	C/T
5854	RP11 62 M	Exon 2–3 deletion c.(?_1)_(238+1_239-1)del	3/3	C/T
1705	RP11 62 F	Exon 2–3 deletion c.(?_1)_(238+1_239-1)del	3/3	C/T
**Families 5–8**				
1175	RP11 36 M	Exon 9–14 deletion c.(855+1_?)del	3/3	C/T
1164	RP11 61 F *	Exon 2–8 deletion c.(−9+1_−8-1)_(855+1_856-1)del	3/3	C/T
1473	RP11 69 M	c.−9+1G>T	3/3	C/T
1708	RP11 70 F	c.527+1G>T	3/3	T/T

* iPSC generated.

## Data Availability

The data presented in this study are available on request from the corresponding author.

## Supplementary Materials

The following are available online at https://www.mdpi.com/article/10.3390/genes12101542/s1, Supplementary Figure S1: Retinal Organoid Differentiation, Supplementary Figure S2: Retinal Pigment Epithelium Differentiation, Supplementary Table S1: List of primers used in this study, Supplementary Table S2: List of antibodies used in this study.
