# Supplementary material for: Determinants of Disease Penetrance in PRPF31-Associated Retinopathy
Source: Genes (Basel). 2021 Sep 28;12(10):1542. doi: 10.3390/genes12101542 (PMC8535263; doi:10.3390/genes12101542)
Supplement: Supplementary file 1 [file genes-12-01542-s001.zip › genes-1388539-supplementary.pdf]

Supplementary Materials

Determinants of Disease Penetrance in PRPF31-Associated Retinopathy

Samuel McLenachan, Dan Zhang, Janya Grainok, Xiao Zhang, Zhiqin Huang, Shang-Chih Chen, Khine Zaw, Alanis Lima, Luke Jennings, Danial Roshandel, Sang Yoon Moon, Rachael C. Heath Jeffery, Mary S. Attia, Jennifer A. Thompson, Tina Lamey, Terri McLaren, John De Roach, Sue Fletcher, Fred K. Chen

Supplementary Figure S1: Retinal Organoid Differentiation
Supplementary Figure S2: Retinal Pigment Epithelium Differentiation

Supplementary Table S1: List of primers used in this study
Supplementary Table S2: List of antibodies used in this study

Supplementary Figure S1

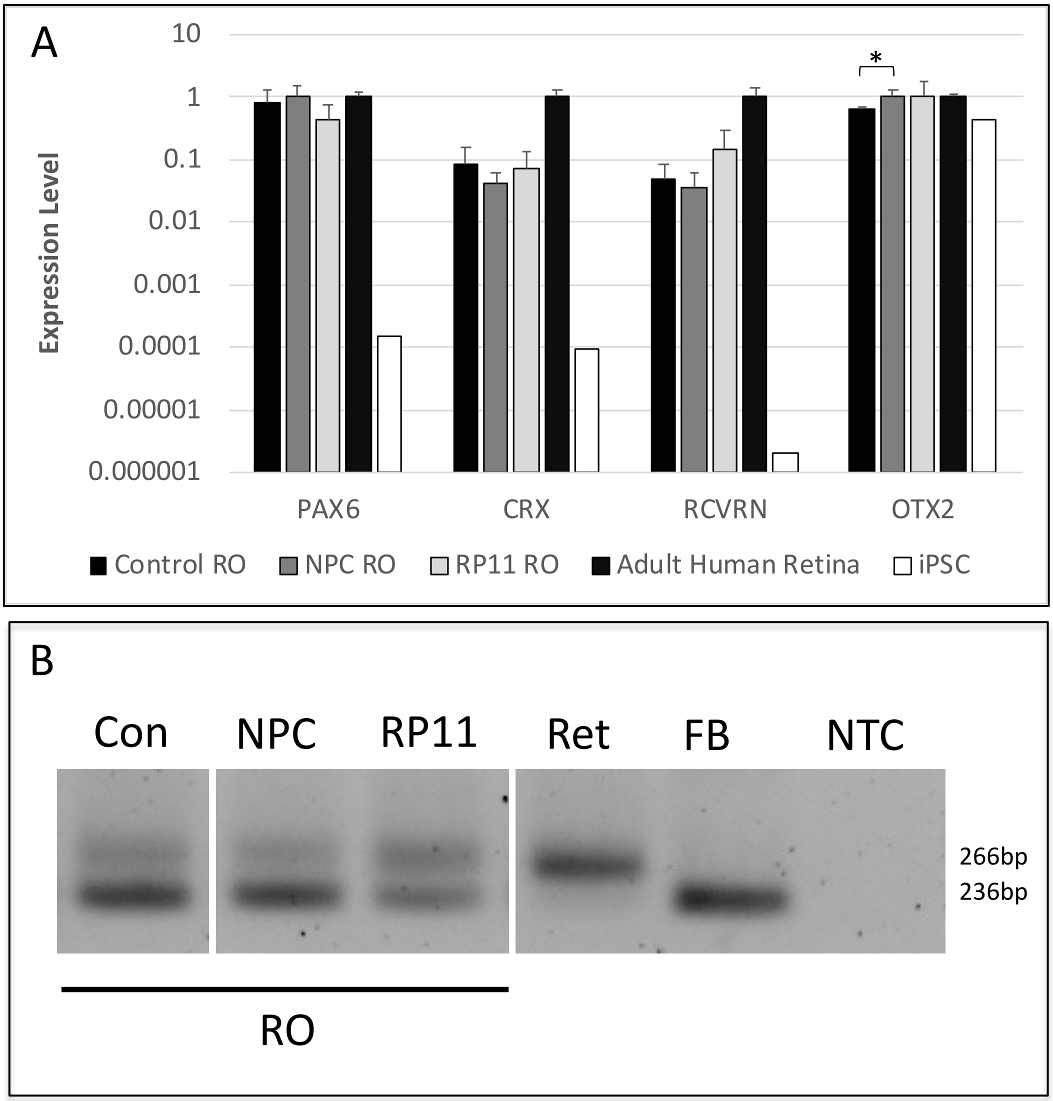

Supplementary Figure S1. Retinal Organoid Differentiation
(**A**) Expression of the retinal markers *PAX6*, *CRX* , *RCVRN* and *OTX2* was measured in retinal organoids (RO) derived from control, non penetrant carrier (NPC) and RP11 patient (c.1205C>A) iPSC lines by qPCR. Adult human retinal tissues (3 donors, aged 22-64) and undifferentiated iPSC were included as controls. Gene expression was normalized to *GAPDH* and expressed as fold-change compared with values obtained from adult human retina. Mean values were calculated from 3-4 retinal organoids from each genotype. Error bars indicate standard deviation. Statistical significance was calculated using an unpaired t-test (*$p<0.05$). All retinal markers showed upregulation relative to undifferentiated iPSC. No significant differences in *PAX6*, *CRX* and *RCVRN* gene expression were detected between RO of different genotypes. *OTX2* expression was significantly increased (1.6-fold) in NPC RO. (**B**) BBS8 transcripts were amplified in control (Con), NPC and RP11 patient (c.1205C>A) retinal organoids after 6 months of differentiation by RT-PCR. Adult human retina (Ret) and fibroblasts (FB) expressed the retinal specific (266bp) and non-retinal (236bp) *BBS8* transcripts, respectively. Both the retinal specific band and the non-retinal band were detected in retinal organoids, confirming retinal specific *BBS8* transcripts were present.

Supplementary Figure S2A

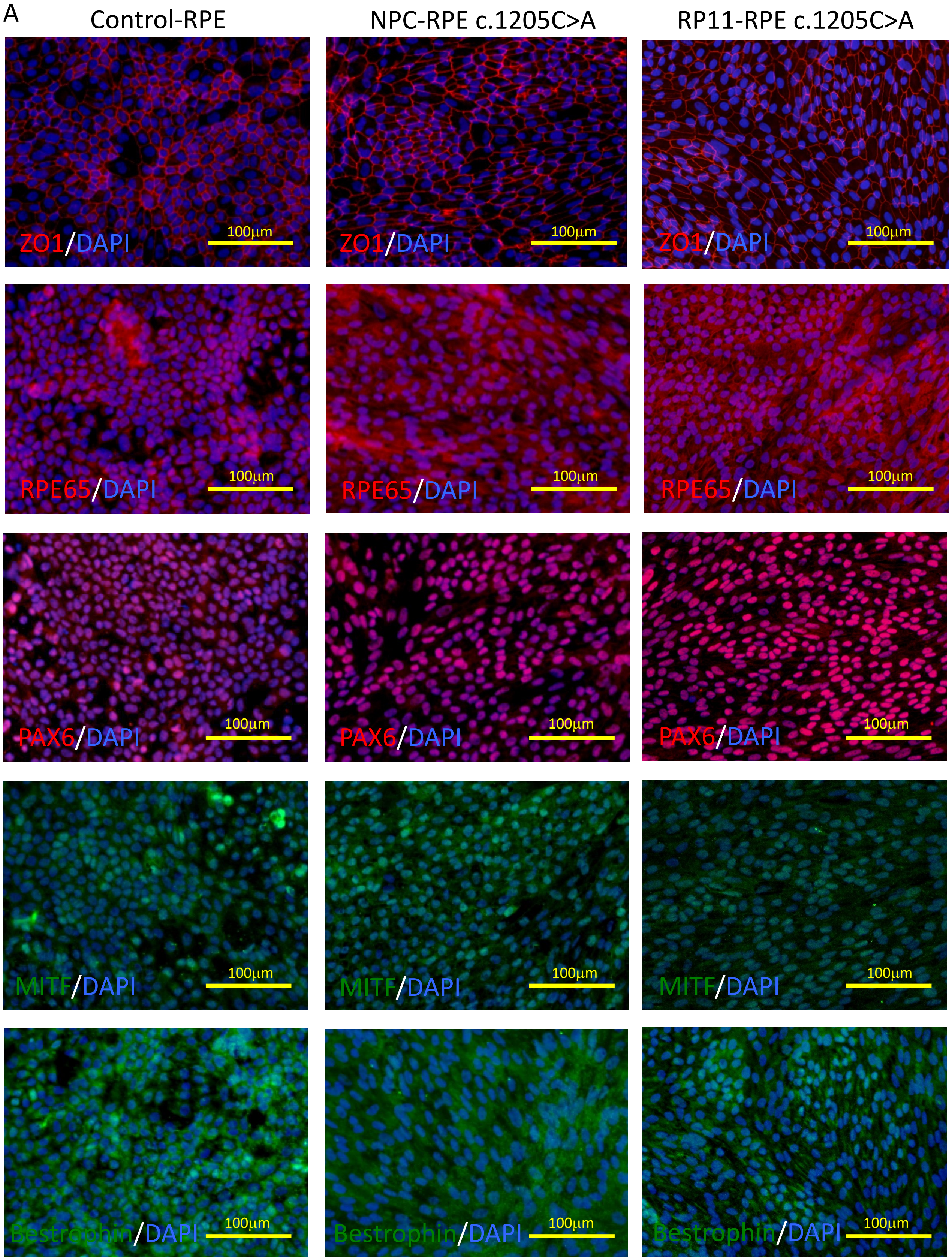

Supplementary Figure S2. A Retinal Pigment Epithelium Differentiation

A: Merged fluorescence micrographs demonstrating expression of the RPE markers ZO1, RPE65, PAX6, MITF and bestrophin in RPE derived from control, non-penetrant carrier (NPC c.1205C>A) and RP11 patient (c.1205C>A) iPSC lines. Cell nuclei were stained with DAPI (blue signal).

Supplementary Figure S2B

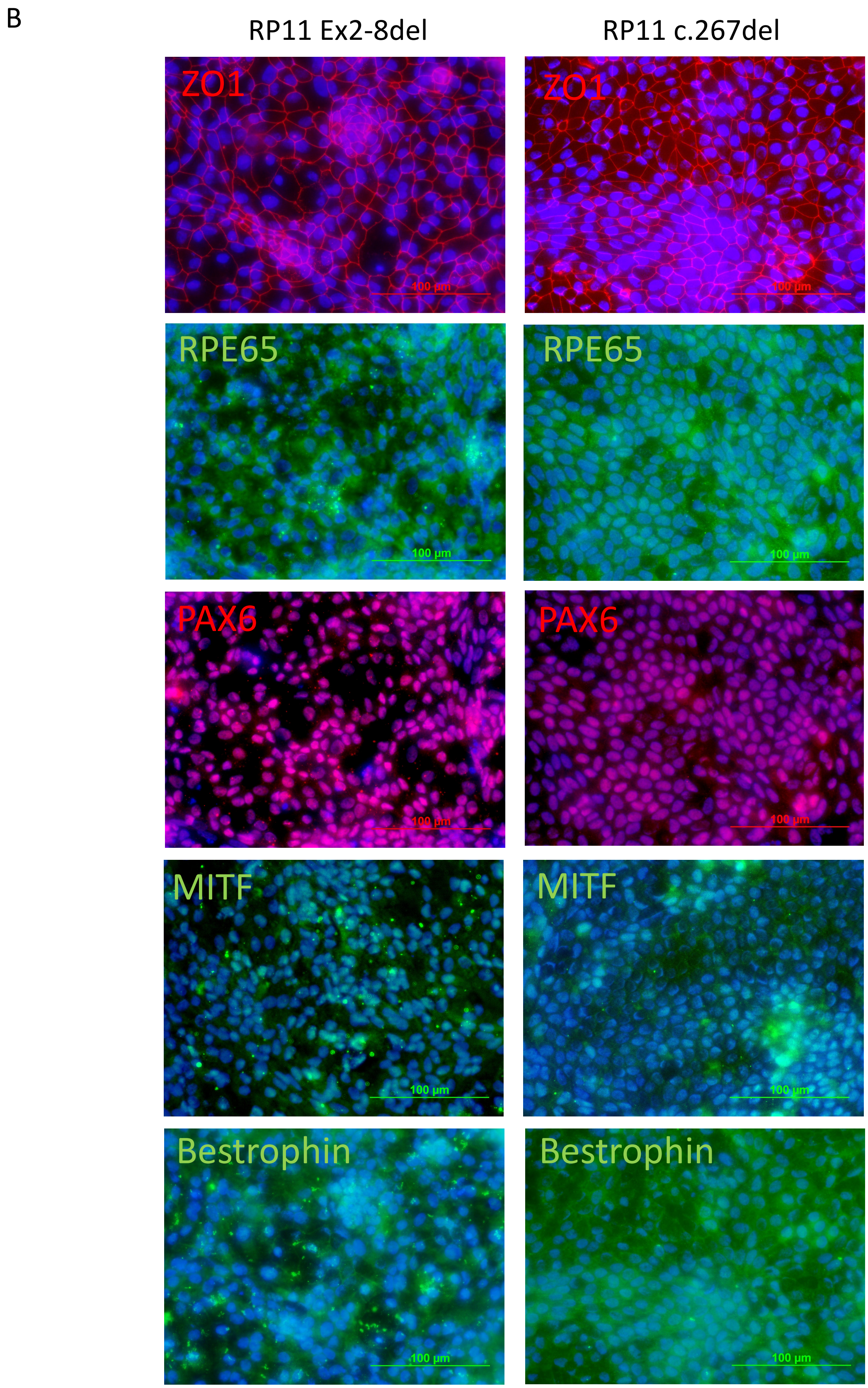

Supplementary Figure S2. B: Retinal Pigment Epithelium Differentiation
A: Merged fluorescence micrographs demonstrating expression of the RPE markers ZO1, RPE65, PAX6, MITF and bestrophin in RPE derived from RP11 patients carrying a deletion of exons 2-8 or the c.267del variant in *PRPF31*. Cell nuclei were stained with DAPI (blue signal).

Supplementary Figure S2C-D

C

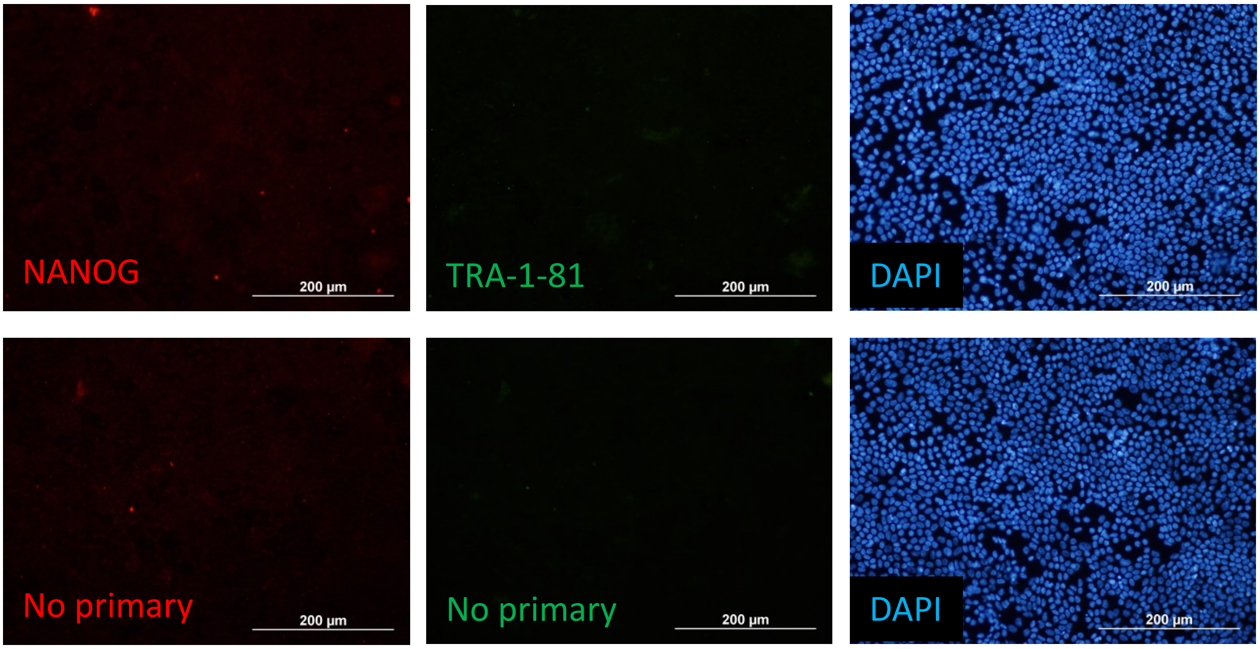

D

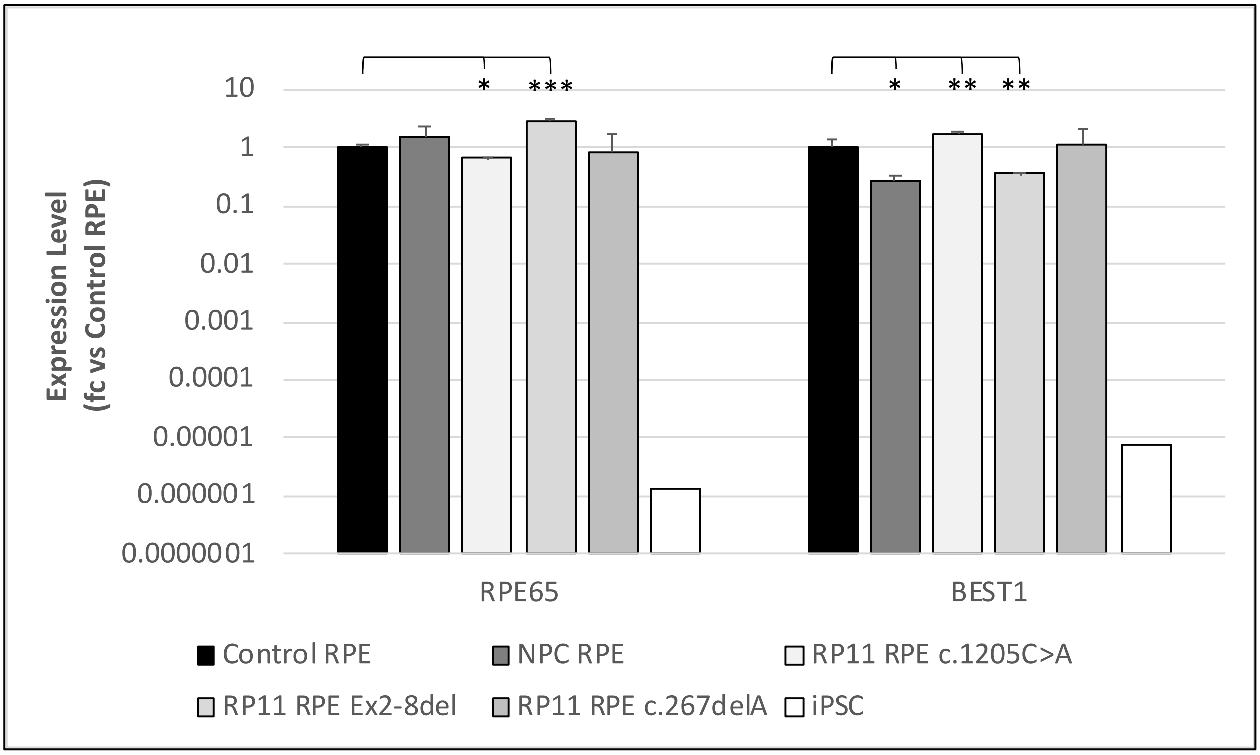

Supplementary Figure S2. C-D: Retinal Pigment Epithelium Differentiation

C: Isogenic control antibodies for the pluripotency markers NANOG (red signal) and TRA-1-81 (green signal) showed no staining in iPSC-derived RPE. RPE cells treated with secondary antibodies (No primary) alone showed no background staining. Cell nuclei were stained with DAPI (blue signal). **D:** Gene expression was measured in RPE derived from iPSC by qPCR. Undifferentiated iPSC were included as a negative control. Gene expression was normalized to *GAPDH* and expressed as fold-change compared with control RPE. Mean expression levels were calculated across samples from 3 culture wells. Error bars indicate standard deviation. Statistical significance was calculated using an unpaired t-test (*p<0.05, **p<0.01 ***p<0.001). The RPE markers *RPE65* and *BEST1* were strongly upregulated in RPE derived from the RP11 patients (RP11 RPE), the non-penetrant carrier (NPC RPE) and a control subject (Control RPE) compared with undifferentiated iPSC. *RPE65* expression was not significantly different from control RPE in NPC and RP11 c.267delA RPE, but was decreased in RP11 c.1205C>A RPE and increased in RP11 Ex2-8del RPE. Compared with control RPE, BEST1 expression was significantly reduced in RPE from the NPC and the RP11 Ex2-8del patient, increased in RPE from the RP11 c.1205C>A patient and unchanged in RPE from the RP11 c.267delA patient.

Supplementary Table S1. List of primers used in this study

Target	Forward Primer	Reverse Primer
GAPDH	GTCTCCTCTGACTTCAACAGCG	ACCACCCTGTTGCTGTAGCCAA
PRPF31	ACGAGCTGAACATCATCCATAAG	CTTGACCGTGCGGATGTAAT
CNOT3	CATCCGCAAGATCAAGGACGAC	TCTCATCCTCCATGTGGCTGTG
PRPF4	GGTAATGTGGCATCCTTCAGGAC	GATGCAGGATCTCCTCTTGAGC
PRPF6	GTGTGGAGATCAACCGTGAGCA	CCATCCAGGTATGCTTCCGATC
PRPF8	AACTGGTATCGGGAGCATTG	TCAGGGCATTCAGCACATAG
SNRNP200	TGACCATCACGCCAGACTTCCA	AGAATCACCTCGCTGTCCACATC
PAX6	CTGAGGAATCAGAGAAGACAGGC	ATGGAGCCAGATGTGAAGGAGG
CRX	CCAGTGTGGATCTGATGCACCA	GGTACTGGGTCTTGGCAAACAG
RCVRN	CCAGAGCATCTACGCCAAGTT	CCGTCGAGGTTGGAATCGAAG
OTX2	GGAAGCACTGTTTGCCAAGACC	CTGTTGTTGGCGGCACTTAGCT
BBS8	TGGCCTGGAGCTATTTTAGG	TCCAGGGAGTTTCAAAGACG
RPE65	TTTGGCACCTGTGCTTTCCCAG	GTTGGTCTCTGTGCAAGCGTAG
BEST1	TGCCAACCTGTCAATGAAGGCG	TCCAGTCGTAGGCATACAGGTG
OCT4	CCTGAAGCAGAAGAGGATCACC	AAAGCGGCAGATGGTCGTTTGG
CNOT3 rs4806718	AAGGCCCTAAAGAAGCAGTC	GTTCTCTGAGGACAAGGACCT

Supplementary Table S2. List of antibodies used in this study

Antibody	Host species	Dilution	Catalogue No. / Source
anti-OCT4 antibody	Mouse	1 : 100	60093 / StemCell
anti-Nanog antibody	Rabbit	1 : 200	ab21624 / Abcam
anti-Sox2 antibody	Rabbit	1 : 50	48-1400 / Invitrogen
anti-TRA-1-81 antibody	Mouse	1 : 100	60065 / StemCell
anti-ZO-1 antibody (1A12), AlexaFluor594	Mouse	1 : 200	339194 / Invitrogen
anti-RPE65 antibody (E-5)	Mouse	1 : 200	SC-390787 / Santa Cruz
anti-CRALBP antibody (B2)	Mouse	1 : 100	ab15051 / Abcam
anti-PAX6 antibody (AD2.38)	Mouse	1 : 100	ab78545 / Abcam
anti-MITF antibody (C5/D5)	Mouse	1 : 100	X2398M / Invitrogen
anti-Bestrophin	Rabbit	1 : 200	ab14928 / Abcam
anti-ARL13B	Rabbit	1:100	17711-1-AP / Proteintech
anti-Rabbit IgG-AlexaFluor488	Goat	1:500	A-11008 / Molecular Probes
anti-mouse IgG-AlexaFluor546	Goat	1:500	A-11003 / Molecular Probes
